# Terrestrial and Marine Foraging Strategies of an Opportunistic Seabird Species Breeding in the Wadden Sea

**DOI:** 10.1371/journal.pone.0159630

**Published:** 2016-08-15

**Authors:** Stefan Garthe, Philipp Schwemmer, Vitor H. Paiva, Anna-Marie Corman, Heino O. Fock, Christian C. Voigt, Sven Adler

**Affiliations:** 1 Research & Technology Centre (FTZ), University of Kiel, Hafentörn 1, D-25761 Büsum, Germany; 2 Institute of Marine Research (IMAR) and Marine and Environmental Sciences Centre (MARE), Department of Life Sciences, University of Coimbra, 3004-517 Coimbra, Portugal; 3 Thünen-Institute of Sea Fisheries, Palmaille 9, D-22767 Hamburg, Germany; 4 Department of Evolutionary Ecology, Leibniz Institute for Zoo and Wildlife Research, Alfred-Kowalke-Str. 17, 10315 Berlin, Germany; 5 Swedish University of Agricultural Sciences, Umeå, Sweden; Centre National de la Recherche Scientifique, FRANCE

## Abstract

Lesser black-backed gulls *Larus fuscus* are considered to be mainly pelagic. We assessed the importance of different landscape elements (open sea, tidal flats and inland) by comparing marine and terrestrial foraging behaviours in lesser black-backed gulls breeding along the coast of the southern North Sea. We attached GPS data loggers to eight incubating birds and collected information on diet and habitat use. The loggers recorded data for 10–19 days to allow flight-path reconstruction. Lesser black-backed gulls foraged in both offshore and inland areas, but rarely on tidal flats. Targets and directions were similar among all eight individuals. Foraging trips (n = 108) lasted 0.5–26.4 h (mean 8.7 h), and ranges varied from 3.0–79.9 km (mean 30.9 km). The total distance travelled per foraging trip ranged from 7.5–333.6 km (mean 97.9 km). Trips out to sea were significantly more variable in all parameters than inland trips. Presence in inland areas was closely associated with daylight, whereas trips to sea occurred at day and night, but mostly at night. The most common items in pellets were grass (48%), insects (38%), fish (28%), litter (26%) and earthworms (20%). There was a significant relationship between the carbon and nitrogen isotope signals in blood and the proportional time each individual spent foraging at sea/land. On land, gulls preferentially foraged on bare ground, with significantly higher use of potato fields and significantly less use of grassland. The flight patterns of lesser black-backed gulls at sea overlapped with fishing-vessel distribution, including small beam trawlers fishing for shrimps in coastal waters close to the colony and large beam-trawlers fishing for flatfish at greater distances. Our data show that individuals made intensive use of the anthropogenic landscape and seascape, indicating that lesser black-backed gulls are not a predominantly marine species during the incubation period.

## Introduction

Information on the foraging behaviour and distribution patterns of seabirds are crucial for understanding the basic aspects of their ecology. Such information is important for identifying the nature and magnitude of interactions with populations of prey species (e.g. [1–3]), as well as for assessing potential conflicts between seabirds and humans in terms of their responses to anthropogenic habitat changes, such as offshore wind turbines and fisheries [4, 5]. To study this, individual approaches are important, particularly in birds that are not restricted to offshore regions but which also use the entire coastal landscape, including intertidal areas and terrestrial sites, and thus exhibit complex foraging strategies.

Gulls (*Larus spp*.) belong in this category. Previous studies based on visual observations demonstrated that black-headed gulls (*Larus ridibundus*) exhibited a dual foraging strategy, using marine habitats as well as coastal mainland sites [6]. The lesser black-backed gull (*Larus fuscus*) has formerly been described as a species that forages mainly in marine waters [7–9]. However, recent alterations in farming practices have led to more open-soil fields associated with more-intensive corn (*Zea mays*) production, while an intensification of mechanical soil treatment potentially increases food availability for gulls in coastal mainland sites, particularly during the breeding period [6, 10, 11]. This species makes extensive use of these foraging options and shows frequent foraging flight into inland areas [12, 13].

We therefore studied this dual-habitat foraging strategy in breeding lesser black-backed gulls at one of the largest colonies in Germany by various technological, observational and analytical means. We equipped incubating gulls with GPS loggers, which enabled us to record data almost constantly during a pre-defined period with a relatively high spatial resolution (see also [14]). We analysed the data to determine how foraging-trip characteristics and area-restricted searching (ARS; e.g. [15, 16]) differed between terrestrial and marine foraging habitats and among individuals, as well as to elucidate temporal patterns in this dual foraging strategy. Intensive night-time feeding at fishing vessels by lesser black-backed gulls has been described previously [17] and we used Vessel Monitoring System (VMS) data to relate ARS to the abundance of fishing vessels [18, 19, 20].

GPS data have a relatively high spatial resolution. It was therefore possible to relate the habitat choice of gulls at terrestrial sites to particular crop types or pastures, allowing preferences for particular farming practices to be analysed (see [21] for an approach using visual observation methods). Identification of prey remains and stable isotope analysis (SIA) of blood from lesser black-backed gulls were combined with foraging-trip data to provide information on individual habitat selection. We also discuss the advantages and disadvantages of marine and terrestrial foraging with particular focus to the availability of foraging habitats, prey availability and foraging efficiency, and address the issue of whether increased terrestrial foraging might reflect important changes in the marine environment.

## Material and Methods

### Work at study colony

**Fig 1:**
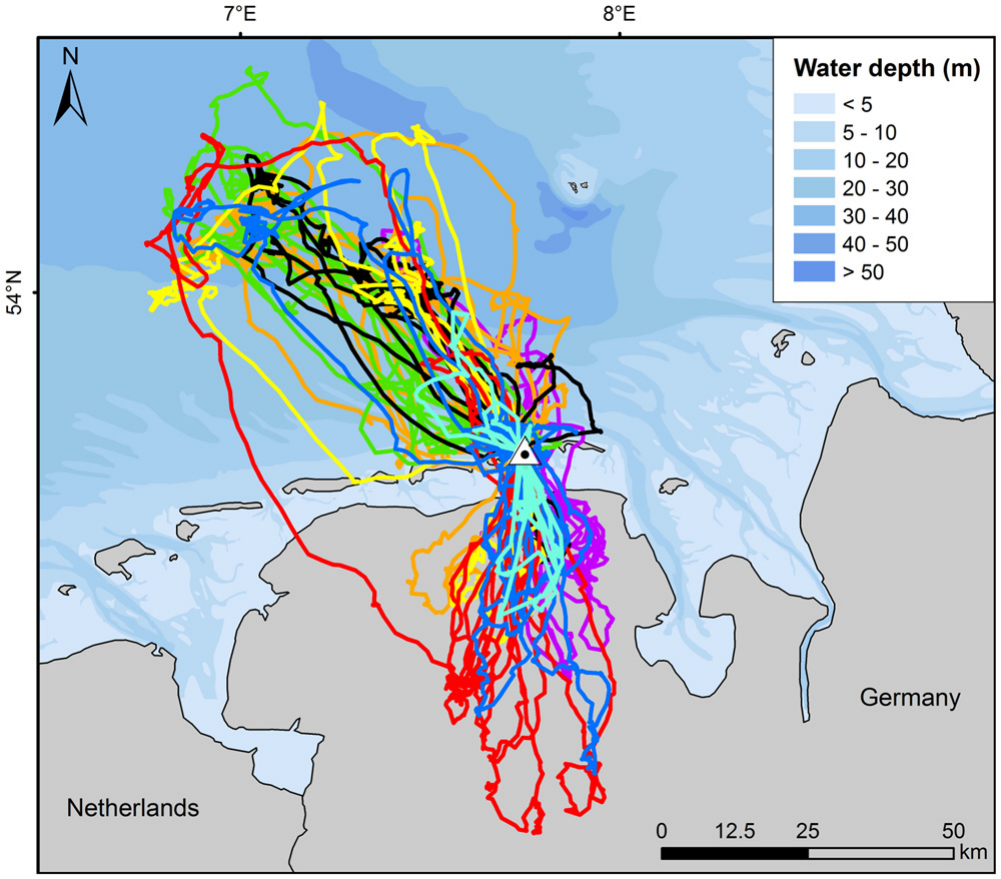
Flight tracks of eight lesser black-backed gulls breeding on Spiekeroog. Each colour represents all tracks from one individual. The study period was from 17 May to 4 June 2010. The location of the colony on the island of Spiekeroog in the south-eastern Wadden Sea is indicated by a triangle. Areas with water depth <5 m represent the maximum extension of tidal flats during low tide.

Fieldwork was carried out on Spiekeroog (53.78° N, 7.76° E; Fig 1), East Frisian Islands, in the southern North Sea, in Germany’s second-largest colony of lesser black-backed gulls (approximately 7–8,000 pairs). A sub-colony of 300–500 pairs located between the dunes and saltmarshes was selected. Nine incubating adults were captured using a walk-in trap set above the nest on 16 and 17 May 2010. They were ringed and colour-marked, and equipped with GPS data loggers. Seven birds were recaptured between 27 and 30 May 2010, and the other two birds on 18 June 2010. After recapture, all birds were weighed to the nearest 1 g and a maximum of 1 ml of blood was sampled from the cutaneous ulnar vein for SIA.

All animal work has been conducted according to relevant national and international guidelines. All sampling procedures and manipulations were reviewed or specifically approved as part of obtaining the field permit. Access to land was approved by the National Park Administration of the Wadden Sea National Park of Lower Saxony, Germany, field procedures and animal manipulations were approved by the Lower Saxony State Office for Consumer Protection and Food Safety, Germany (file number: 33.9-42502-04-09/1635).

### GPS data loggers

mGPS-2 data loggers (Earth and Ocean Technologies, Kiel, Germany) were placed in a box measuring 46.5 mm × 32 mm × 18.5 mm (maximum length × width × height). The total weight was 23 g, which represented about 2.8–3.7% of the bird’s body mass (623–834 g; mean, 736 g). The box was attached to the base of the four innermost tail feathers with Tesa^®^ tape. The devices were set to record date, time, position and flight speed every 3 min after the last satellite uplink (usually took 4–8 s). Eight of the nine loggers recorded regularly over a period of 10–19 days (mean, 14 days), but one device malfunctioned. The recovered data thus covered on average half of the incubation period (incubation period 26–31 days; [22]). Overall, the data loggers recorded from 17 May to 4 June 2010. All GPS positions were checked for plausibility.

### Instrumentation

The GPS loggers had no apparent effects on the birds. All nine birds that were equipped with data loggers were successfully recaptured. The possible loss of the first clutch in two birds happened several days after deploying the devices, while the field investigators were absent; the birds had been observed to be incubating up to that time. Clutch losses in gulls and other seabird species are relatively common, particularly during early incubation. One captured individual had also been part of a previous, smaller study in 2009. Changes in body masses of the seven individuals between first capture and recapture showed increases in three and decreases in three, while the body mass of one individual remained almost constant. The mean body mass at initial capture was 764 g, and that at recapture was 765 g. These results suggest that the devices had no strong negative effect on the foraging behaviours of the tracked animals or on the study outcome.

### Foraging-trip analysis

Foraging trips were defined as all trips targeting the open sea, the mainland, or other areas, but which clearly led away from the colony, as confirmed by spatial analyses using ArcGIS 10.0 (ESRI). No signs of foraging activities were detected by visual observations near the colony, neither in the dunes nor on the beaches. All statistical analyses were performed using the open-source software R 3.0.0 [23]. The significance level was set at p < 0.05.

The foraging range was defined as the direct distance between the nest and the most distant point of a foraging trip. Foraging range and total distance flown were calculated using the R-package *trip* 1.1–15 [24]. The straightness index was derived from [25] (see also [26]), with (foraging range)×2×total distance flown^-1^. This results in a maximum value of 1 for a completely straight flight from the colony to the most distant point and back, and gives increasingly smaller values for less directed, more tortuous flights.

We set up four different models to analyse the effects of trip destination (i.e. sea or inland) on: (1) foraging-trip duration; (2) foraging range; (3) total distance flown; and (4) straightness index (response variables). Mixed trips (i.e. trips targeting both sea and land) and trips to Spiekeroog island were excluded from the analysis, leaving 99 foraging trips for analysis. Explanatory variables were log-transformed when necessary. All models were calculated with a Gaussian error distribution. Linear mixed models (LMMs; [27]) were analysed using functions within the R packages *lme4* [28] and *lmerTest* [29]. All LMMs included trip identity nested within the individual as a random term to account for pseudo-replication.

Human activities influencing the foraging behaviour of gulls (fishing, agriculture) may vary over a weekly cycle because of human activity schedules, and the relative uses of land and sea areas were therefore analysed for each day of the week. The proportions of positions over the mainland and over the sea were calculated for each individual on each weekday, and then averaged (including standard errors) over all eight individuals.

### Foraging behaviour

Hotspots of foraging activity for each individual were identified using the First Passage Time (FPT) technique [15]. FPT is defined as the time required for an animal to pass through a circle with a given radius *r*. By moving this circle along the path of the animal, we could obtain a scale-dependent measure of search effort, and therefore the behavioural response of an individual in the environment. Because marine top predators usually forage in patchy and hierarchical environments [30], increases in turning rate and/or decreases in speed along a foraging path should be related to ARS behaviour [31]. ARS will then appear as an individual reaction to changes in resource availability and distribution, by increasing the residence time in the productive patch [15].

In-water positions are usually associated with very small-scale ARS zones (< 100 m diameter), which considerably increase the variance in FPT and can camouflage larger-scale ARS zones [16]. To address this problem, we removed bouts of time spent on the water and interpolated locations to obtain a distance interval of 0.1 km for FPT analysis [32]. We considered positions with speed < 3 km h^-1^ as indicating resting or preening behaviour on the water or inland, based on the frequency distribution of all measured speeds. Following the recommendations of [32], FPT analysis was performed in two steps: (1) to detect large-scale ARS, we ran the analysis for the whole path, estimating the FPT every 1 km for a radius *r* from 1–50 km; (2) to detect small spatial-scale events, we ran FPT analysis every 0.1 km for an *r* of 0.1–10 km. The plot representing variance in log (FPT) as a function of *r* allowed us to identify the ARS scales by peaks in the variance (S1 Fig). In this calculation, FPT was log-transformed to make the variance independent of the magnitude of the mean FPT [15]. Then, the decision on which zone should be defined as an ARS or not along each foraging track was made setting a threshold value in FPT. Such threshold was set after inspecting the frequency distribution of FPT values, at the r of maximum variance in FPT previously identified for each trip. It was also possible to locate where the bird entered an ARS zone and the time spent in that area by plotting FPT values, where a peak of FPT occurred as a function of time since departure from the colony.

Foraging strategies of individual birds were measured using the following parameters: (1) number of ARS zones per trip, representing the investment in ARS within each foraging foray; (2) scale of ARS zone (radius of ARS in km), as the scale at which birds increased their search effort; (3) maximum FPT (h), as the maximum time of residence within an ARS zone; and (4) distance of ARS from colony (km), as the distance between the colony and the main ARS zone. To reach land from their breeding colony, the lesser black-backed gulls needed to travel at least 7.8 km (direct distance). We therefore compared ARS behaviours of birds foraging over sea and over land by selecting sea foraging excursions at least 7.8 km direct distance from the colony. This excluded 12 of the 99 recorded trips, but no ARS behaviour was exhibited in any of those trips (i.e. excursions were normally too short in duration for the ARS behaviour to be detected). Eight trips with a mixture of sea and land foraging destinations were also excluded from the analysis, yielding a final sample size of 79 foraging trips for analysis.

Similar to the analyses of foraging trips, LMMs were used with foraging-strategy parameters as response variables to assess differences in foraging behaviours between land and sea destinations. The best procedure to apply under the LMM framework was selected based on the decision tree for LMM fitting and inference and advice from [33]. The response variables to test the previous hypothesis were therefore: (1) scale of ARS; (2) maximum FPT while within the ARS zones; and (3) distance from ARS to colony. All LMMs included trip identity nested within the individual as a random term to account for pseudo-replications.

### Diet collection and analysis

Dietary information was derived from pellets and SIA. Fifty pellets were collected in the colony on 28 May 2010; none of the pellets could be directly allocated to the logger birds. They were analysed to the lowest possible taxon, following standard procedures (e.g. [8]).

SIA is commonly used to assessing the trophic ecology of seabirds (e.g. [34]). Isotopic fractionation means that isotopic signatures vary among prey and consumers. This variation can be measured in different tissues, providing information on diets and habitat selection [34]. Because isotopic turnover rates differ among different tissues, SIA provides an integrated measure of diet over different time scales. Plasma is known to represent dietary information for about 3 days prior to sampling, while red blood cells integrate foraging behaviour over 3–4 weeks (e.g. [35, 36]). Both components can thus be used to study medium- and short-term foraging behaviours in gulls [18, 37]. Trophic levels (nitrogen) and foraging domains (sea vs. land; carbon) in individual gulls were investigated by SIA. Blood samples were centrifuged shortly after sampling, separating plasma and red blood cells, and subsequently frozen. The samples were further analysed at the stable isotope laboratory of the Leibniz Institute for Zoo and Wildlife Research (IZW) in Berlin (Germany). The samples were dried at 60°C to constant mass and then weighed in tin cups. Stable isotope ratios in tissue samples were analysed using an isotope ratio mass spectrometer (Delta V Advantage; Thermo Finnigan, Bremen, Germany) connected to an elemental analyser (Flash elemental analyser; Thermo Finnigan) via a ConFlo III (Thermo Finnigan). We used 0.4 mg samples combusted under chemically-pure helium gas in the elemental analyser. By convention, we expressed stable isotope ratios using the δ notation in relation to an international standard (Vienna-PeeDee Belemnite for carbon and air N_2_ for nitrogen), and the unit of measurement as ‰ deviations from the respective international standards. We calculated δ values using the following equation: δX = [(R_sample_ ⁄R_standard_)– 1] × 1000; where X is the heavy isotope of either carbon (^13^C) or nitrogen (^15^N), and R is the ratio of heavy:light isotopes (^13^C:^12^C or ^15^N:^14^N). The analytical precision was better than 0.13‰ (one standard deviation) for carbon and 0.14‰ for nitrogen isotopes, based on repeated measures of internal protein standards.

Following [38], we used discrimination factors of 2.4‰ for δ^15^N values and 0.7‰ for δ^13^C values to account for the trophic enrichment between prey and bird blood, based on the average of eight estimations from aquatic birds raised on an aquatic diet. Lipid-associated biases on δ^13^C values were reduced by mathematically normalizing plasma δ13C using the following equation for aquatic animals: δ^13^C_normalized_ = δ^13^C − 3.32 + (0.99 × C:N) ([39], following [40]).

Stable isotopes were not analysed for the two birds recaptured in June, because the blood signal would not have matched the period of logger deployment in these cases. We performed linear regressions between the proportions of GPS locations that were recorded at sea with δ^13^C and δ^15^N, respectively, using linear models (LMs). Increasing δ^13^C values indicate a more marine origin of prey while increasing δ^15^N values indicate prey from higher trophic levels (e.g. fish compared with terrestrial invertebrates; e.g. [34, 41]). We therefore expected a positive relationship between δ^13^C and δ^15^N and the time that an individual lesser black-backed gull spent at sea.

### Habitat selection on land

The use of terrestrial habitats (e.g. crop type in agricultural fields) was analysed based on positions obtained from land, excluding the islands. Movement speeds > 10 km h^-1^ were ignored to exclude flight segments. Feeding spots were identified for all individuals and trips based on the requirement that a bird spent at least 30 min in an area of 500 m × 500 m. This procedure was designed to select the most important feeding spots. If the feeding spot was > 500 m × 500 m, either more than one feeding spot was defined, based on the requirements stated above, or the core area was selected. Up to a maximum of 15 feeding spots was selected for each individual to reduce bias due to the different extent of terrestrial foraging. Foraging trips were chosen randomly if more than 15 feeding spots were available. This procedure resulted in the selection of 89 feeding spots that were investigated in terms of their land use, after finishing fieldwork in the colony. To reduce possible biases further, only the most intensively-used feeding spots per trip and individual were selected. The resulting 38 feeding spots were retained for final analysis.

Habitat availability was mapped on the same days that land use was analysed. Transect counts of land use were carried out from a driving car throughout the area including the study gulls’ feeding spots. A total of 1,292 land-use segments were retained for final analysis.

Availability and use of habitats by lesser black-backed gulls were compared by Monte Carlo permutation tests, based on χ^2^ tests with simulated p values based on 10,000 replicates per analysis. These tests were run under R 3.0.0 [23]. Availability and use of habitats by gulls were tested at two levels: plots with ground-covering vegetation were compared with plots with little or no vegetation (corn, potatoes, summer wheat, ploughed soil, construction area, water body); and habitat types were tested individually.

### Distribution of fishing vessels

The importance of fishery discards as foraging targets for lesser black-backed gulls was estimated by analysing the distribution of fishing vessels derived from the VMS (see e.g. [42]) and plotted on a map using ArcGIS 10. VMS data were prepared for an area larger than the foraging area of the gulls, ranging from 6°E to 9°E longitude, and up to 54.45°N latitude. Data were selected for the complete period when the GPS loggers were collecting data, i.e. 17 May to 4 June 2010. Data were analysed for those fisheries that produce discards and hence act as a potential food source for birds. These fleets in decreasing importance comprised small beam trawlers (< 300 HP), large beam trawlers (> 300 HP) and otter board trawlers from Germany and other countries operating in the study area. Data were filtered according to a procedure developed by [42] to exclude periods when fishing vessels were inactive or were commuting between harbours and fishing areas. Data on fishing-boat operations were anonymised such that the resulting dataset included information on date, time, position and fishing gear. Each trawler provides such information at least once every 2 h, and usually more frequently [42].

## Results

### Distribution and foraging-trip patterns

Lesser black-backed gulls visited both offshore and inland areas during their foraging trips. When heading for the open sea, foraging trips were directed in a (north-) northwesterly direction, while those to inland areas were directed in a southerly direction (Fig 1, S2 Fig). Despite relatively large individual variation in headings, the overall targets and directions were similar among the eight individuals. All eight individuals performed trips out to sea, while seven of the eight also carried out trips towards inland areas. From a total of 108 foraging trips recorded for all eight individuals (9–18 per individual), 52 (48.1%) were solely targeted to the sea (0–14 per individual), 47 (43.5%) solely to inland areas (0–12 per individual), seven (6.5%) were mixed (sea + land; 0–3 per individual) and two (1.9%) were targeted to the island (0–1 per individual). Interestingly, very little foraging activity occurred on the tidal flats in the Wadden Sea between the island and the mainland.

Overall, foraging trips lasted for 0.5–26.4 h (n = 108). The mean duration per individual ranged from 4.9–13.8 h, with an overall mean (± standard error; SE) of 8.7 ± 0.9 h. Foraging ranges (maximum distance from the nest) varied widely from 3.0–79.9 km, with individual means of 14.5–48.1 km and an overall mean (± SE) of 30.9 ± 3.7 km. The total distance travelled per foraging trip also ranged widely from 7.5–333.6 km, with individual means of 40.0–160.5 km, and an overall mean (± SE) of 97.9 ± 12.6 km for all individuals. Straightness indices ranged from 0.98–0.30, with individual means of 0.77–0.59, and an overall mean (± SE) of 0.69 ± 0.02.

**Fig 2:**
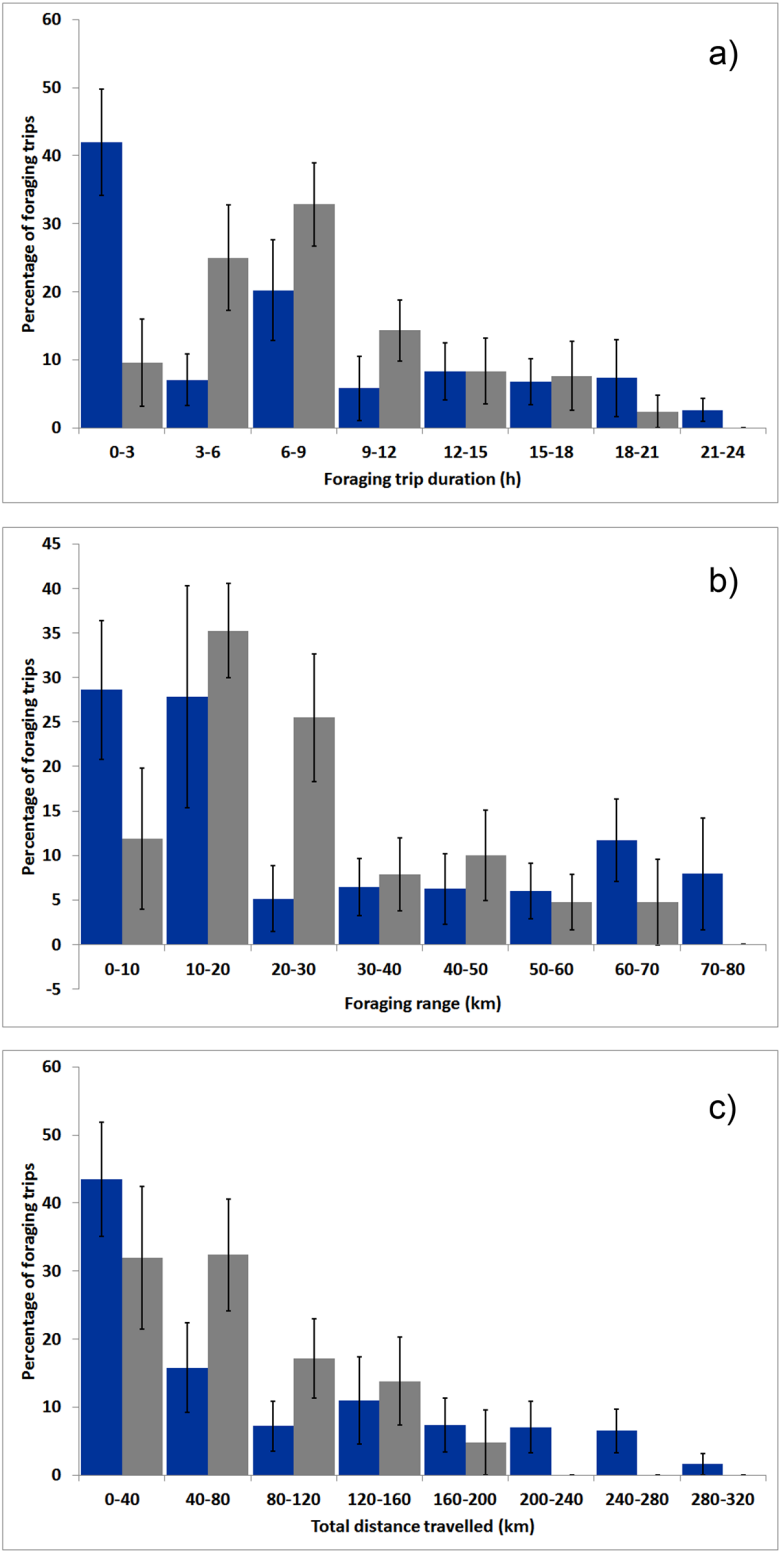
Frequency distribution of (a) foraging-trip duration, (b) foraging range, and (c) total distance travelled for foraging trips targeting the sea (blue bars) and land (grey bars). Bars show the mean percentage over all eight individuals, and the vertical lines represent the standard error.

Although there were some differences in mean values for parameters between trips directed to the sea and those directed to land, these differences were not significant (Table 1). However, variability in foraging trip characteristics was consistently and significantly higher for sea trips (Table 1): (1) sea trips were often of much shorter and longer durations than inland trips (Fig 2a); (2) trips to the open sea often involved very short distances, but also included the longest distances (Fig 2b); and (3) many trips to the open sea involved very short total distances travelled, but a few very extended ones, while the overall flight tracks for inland trips were mostly shorter than 80 km (Fig 2c).

**Table 1:** Comparison of characteristics of foraging trips targeting land or sea in lesser black-backed gulls from Spiekeroog in 2010.

Characteristics	Land	Sea	Linear Mixed Model (LMM)		F-test for variances	
			Χ^2^	*p*	F	*p* (F test)
**Total number of foraging trips**	47	52	-	-	-	-
**Mean (±SE) trip duration (h)**	7.9±4.4	8.0±6.3	0.777	0.378	4.032	**<0.0001**
**Range of trip duration (h)**	1.0–18.2	0.7–21.5	-	-	-	-
**Mean (±SE) foraging range (km)**	25.3±15.0	33.0±26.0	0.030	0.863	2.379	**<0.0001**
**Min. foraging range (km)**	8.7	3.6	-	-	-	-
**Max. foraging range (km)**	65.6	79.9	-	-	-	-
**Mean (±SE) total distance travelled (km)**	73.1±45.1	103.5±87.5	0.003	0.957	0.592	**<0.05**
**Min. total distance travelled (km)**	17.7	8.9	-	-	-	-
**Max. total distance travelled (km)**	188.9	298.6	-	-	-	-
**Mean (±SE) straightness**	0.72±0.12	0.68±0.14	2.757	0.097	0.069	**<0.0001**
**Min. straightness**	0.43	0.40	-	-	-	-
**Max. straightness**	0.98	0.95	-	-	-	-

### Foraging behaviour

**Fig 3:**
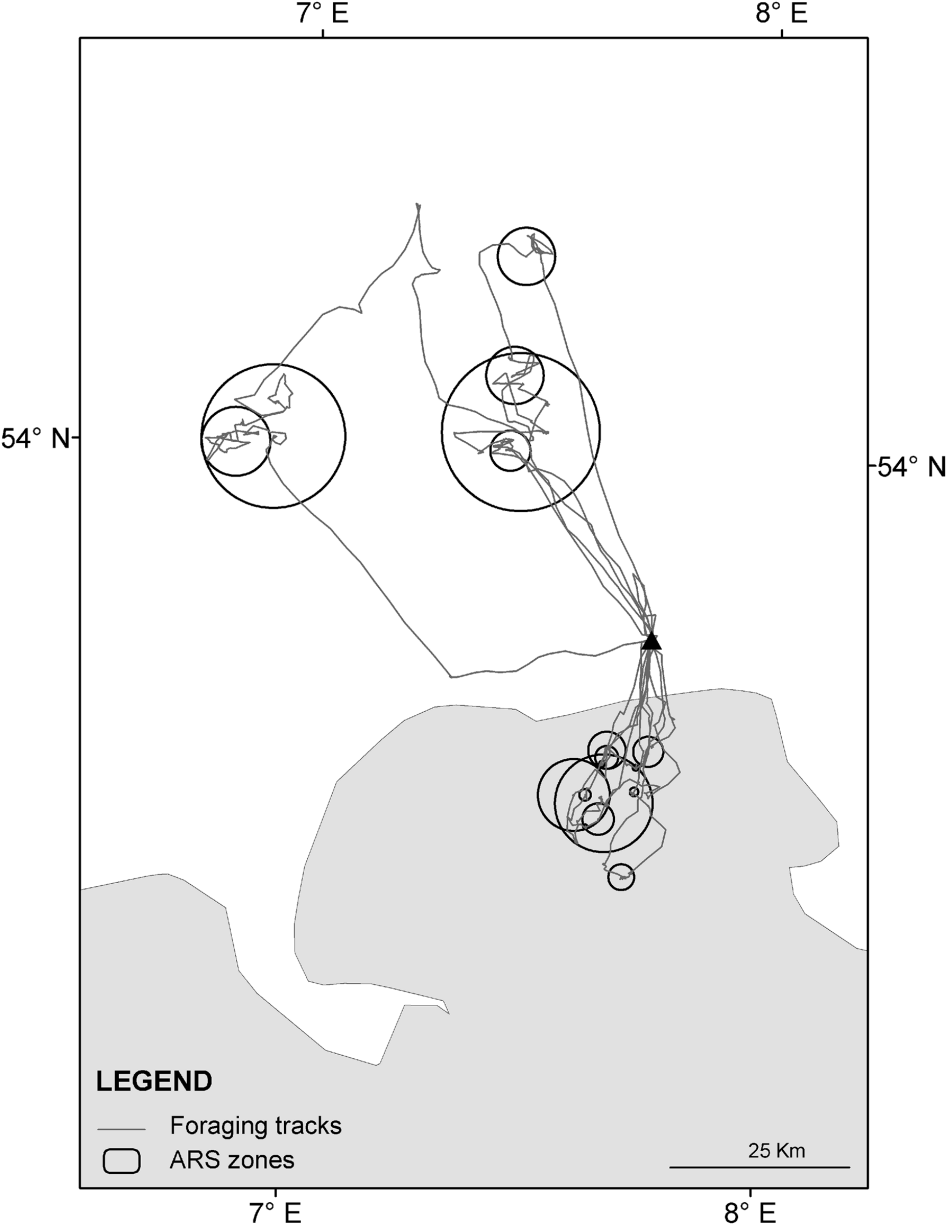
Flight tracks and zones of area-restricted search (ARS) for one lesser black-backed gull breeding on Spiekeroog in 2010. The location of the colony on the island of Spiekeroog is indicated by a triangle.

Analysis of FPT revealed several zones of ARS behaviour per individual (see example trip on Fig 3). Based on this method of measuring foraging behaviour, land and sea trips differed significantly in a variety of parameters (Table 2). Most importantly, the radii of the ARS zones and their distances to the colony were significantly larger at sea (Table 2). This held true for all three scales of ARS (Table 2).

**Table 2:** First passage time analysis of foraging behaviour of lesser black-backed gulls nesting on Spiekeroog in 2010.

Scale	Parameters	Land	Sea	F	*p*
**Colony level**	Total number of foraging trips	43	29	-	-
	Number of ARS zones per trip	1.35±0.65	1.52±0.63	0.03	0.86
	Number of ARS zones	60	43	-	-
	Radii (km)	2.78±3.83	5.85±4.97	16.99	**<0.001**
	Max. FPT (d)	0.18±0.13	0.23±0.18	3.04	0.09
	Mean distance to colony	26.12±14.67	42.39±20.10	17.86	**<0.001**
**3**^**rd**^ **ARS scale**	Number of ARS zones	36	21	-	-
	Radii (km)	1.00±1.31	2.77±2.27	16.71	**<0.001**
	Max. FPT (d)	0.13±0.09	0.14±0.10	0.23	0.63
	Mean distance to colony	24.29±14.30	41.20±21.50	7.72	**<0.01**
**2**^**nd**^ **ARS scale**	Number of ARS zones	13	17	-	-
	Radii (km)	2.37±1.33	6.73±3.00	21.55	**<0.001**
	Max. FPT (d)	0.16±0.06	0.29±0.19	11.27	**<0.01**
	Mean distance to colony	25.40±15.25	41.38±20.01	8.23	**<0.01**
**1**^**st**^ **ARS scale**	Number of ARS zones	11	5	-	-
	Radii (km)	9.11±4.80	15.8±4.55	4.42	**0.05**
	Max. FPT (d)	0.36±0.17	0.39±0.25	0.05	0.82
	Mean distance to colony	32.96±14.52	50.04±15.64	9.31	**0.01**

Comparison of parameters (mean±SE) between foraging trips directed to the open sea and those directed to inland areas, and an overall approach. Differences were tested by Linear Mixed Models (level of significance = 0.05). Significant differences are indicated in bold.

**Fig 4:**
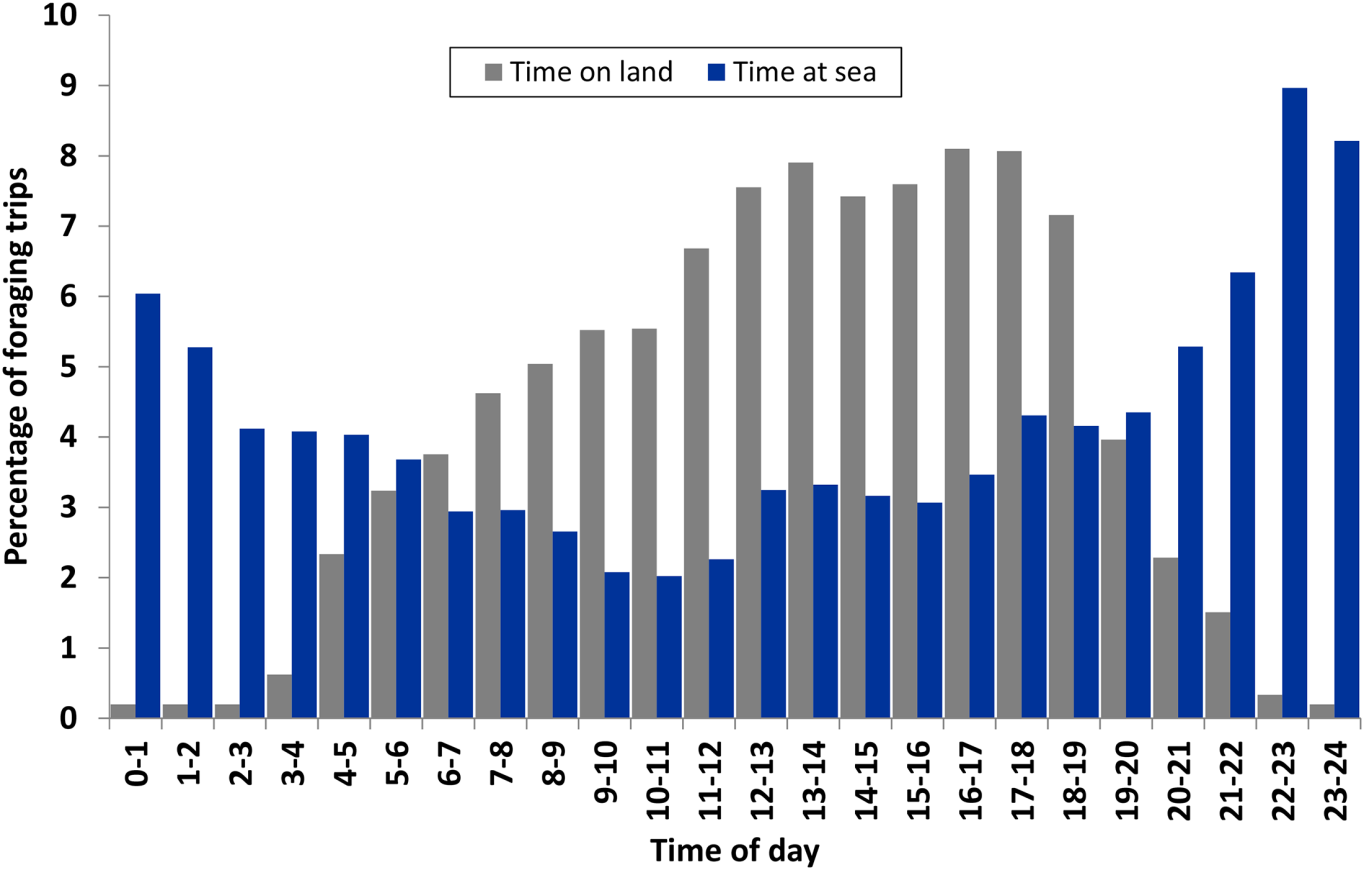
Relative uses of sea and land areas during foraging trips of lesser black-backed gulls breeding on Spiekeroog in 2010 over a 24-h cycle. Bars show the mean percentages of time spent at sea (blue bar) or on land (grey bar) for all eight individuals.

The uses of sea and land areas varied substantially over the daily cycle (Fig 4). Presence at inland areas was closely associated with the period of daylight, with a peak activity between noon and early evening and hardly any data from land at night. The daylight match for terrestrial feeding spots was especially apparent, with use between 03:59 and 21:48 CEST (local average time for sunrise was 05:13 and for sunset was 21:39). Sea areas were used both day and night, but proportionally more during the night hours (Fig 4). Lesser black-backed gulls regularly foraged at sea at night, with periods of intense flight activity and periods with limited flight activity.

The proportional uses of sea and land areas over the 7 days of the week differed on Fridays and Saturdays, with a relatively greater use of sea areas on both these days compared with the rest of the week.

### Diet

The following items were identified in the 50 pellets, in decreasing order of abundance (measured by frequency of occurrence): grass (48%), insects (38%), fish (28%), litter (26%), earthworms (20%), crustaceans (16%), mammals (12%), bivalves (8%), seeds (8%), polychaetes (6%) and eggs (4%). Insects comprised mainly Coleoptera, with Carabidae being the most prominent family, followed by Silphidae and Elateridae. Identified fish species (in decreasing order of abundance) were: grey gurnards (*Eutrigla gurnardus*), cod (*Gadus morhua*), unidentified gurnards, unidentified gadids, scad (*Trachurus trachurus*), and unidentified flatfish. When classified according to their likely origin, all species identified to group level were considered as discards. Identified crustaceans consisted exclusively of swimming crabs (*Liocarcinus spp*.), while in terms of mammals, northern mole (*Talpa europaea)* and common voles (*Microtus arvalis*) could be identified to species level.

**Fig 5:**
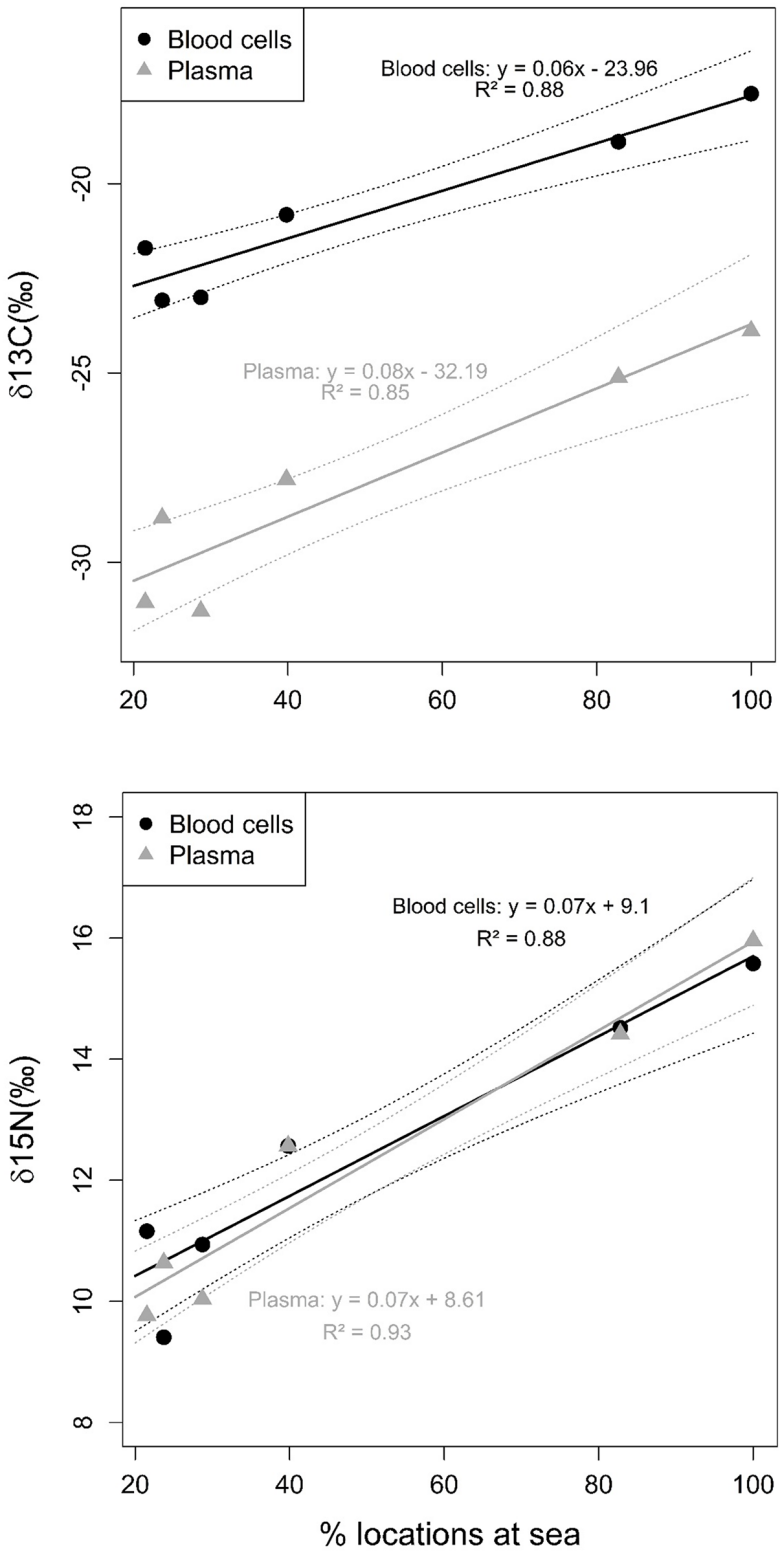
δ^13^C and δ^15^N values from red blood cell and blood plasma in relation to the proportion of foraging spent at sea for the six lesser black-backed gulls for which information was available. One red blood cell and one blood plasma value are given for each individual. Bold lines: regression lines of linear model; dotted lines: 95% confidence interval.

There was a clear relationship between the carbon and nitrogen isotope signals and the proportions of foraging that each individual carried out at sea and on land. According to LM, both δ^15^N and δ^13^C values increased significantly with increasing proportions of foraging at sea (carbon blood cells: t = 6.17, p < 0.01, df = 4; carbon plasma: t = 5.33, p < 0.01, df = 4; nitrogen blood cells: t = 6.01, p < 0.01, df = 4; nitrogen plasma: t = 8.06, p < 0.01, df = 4; Fig 5).

### Terrestrial habitat selection

Gulls preferentially used open ground when foraging on land (Table 3). Grassland, corn, winter wheat, and barley were the most common items in the 1,292 plots. Potato fields were significantly overused in relation to their abundance, while grassland was proportionally underused. There was a non-significant trend for proportional overuse of corn (Table 3).

**Table 3:** Availability of terrestrial habitat types and their uses as feeding spots by lesser black-backed gulls tagged in the colony on Spiekeroog from 17 May to 4 June 2010.

Test	Habitat type	Availability	Feeding spots	Proportional use	Χ^2^	*p*
**Open vegetation vs. ground-covering vegetation**	Overall	1292	38	-	26.00	**<0.001**
	Ground-covering vegetation^a^	1016	17	0.569	-	-
	No/little vegetation^b^	276	21	2.587	-	-
**Various habitats**	Overall	1292	38	-	142.27	**<0.001**
	Grassland^a^	824	17	0.701	5.96	**0.019**
	Potato^b^	12	6	17.000	91.20	**<0.001**
	Waste disposal	0	1	-	-	-
	Corn	248	12	1.645	3.76	0.062
	Water body	2	0	-	-	-
	Summer wheat	3	0	-	-	-
	Rye	15	0	0.000	0.45	1.000
	Construction area	6	1	5.667	3.86	0.161
	Ploughed soil	5	1	6.800	4.97	0.141
	Winter wheat	90	0	0.000	2.85	0.109
	Fallow land	6	0	0.000	0.18	1.000
	Pea	2	0	-	-	-
	Barley	61	0	0.000	1.88	0.258
	Oat	8	0	0.000	0.24	1.000
	Rape	10	0	0.000	0.30	1.000

Proportional use values indicate relative preference (> 1) or avoidance (< 1) of habitats. Significant differences (bold) were tested using Monte Carlo permutation tests (level of significance = 0.05). Tests were only performed for habitat types with an availability >4.

^a^used less than available on average,

^b^used more than available on average.

### Distribution of fishing vessels

VMS signals obtained for the period 17 May to 4 June (n = 25,760 signals) consisted mainly of small beam trawlers (96.3%), with fewer signals for otter board trawlers (2.7%) and large beam trawlers (1.0%). Overall fishing activity did not vary markedly for any fleet over the daily cycle when the whole GPS logger period was considered; thus fishing activity takes place during night and day. Fishing activities were similar from Sundays to Thursdays, but lower on Fridays and Saturdays, with 76% and 56%, respectively, of the mean numbers of signals from Sunday to Thursday.

**Fig 6:**
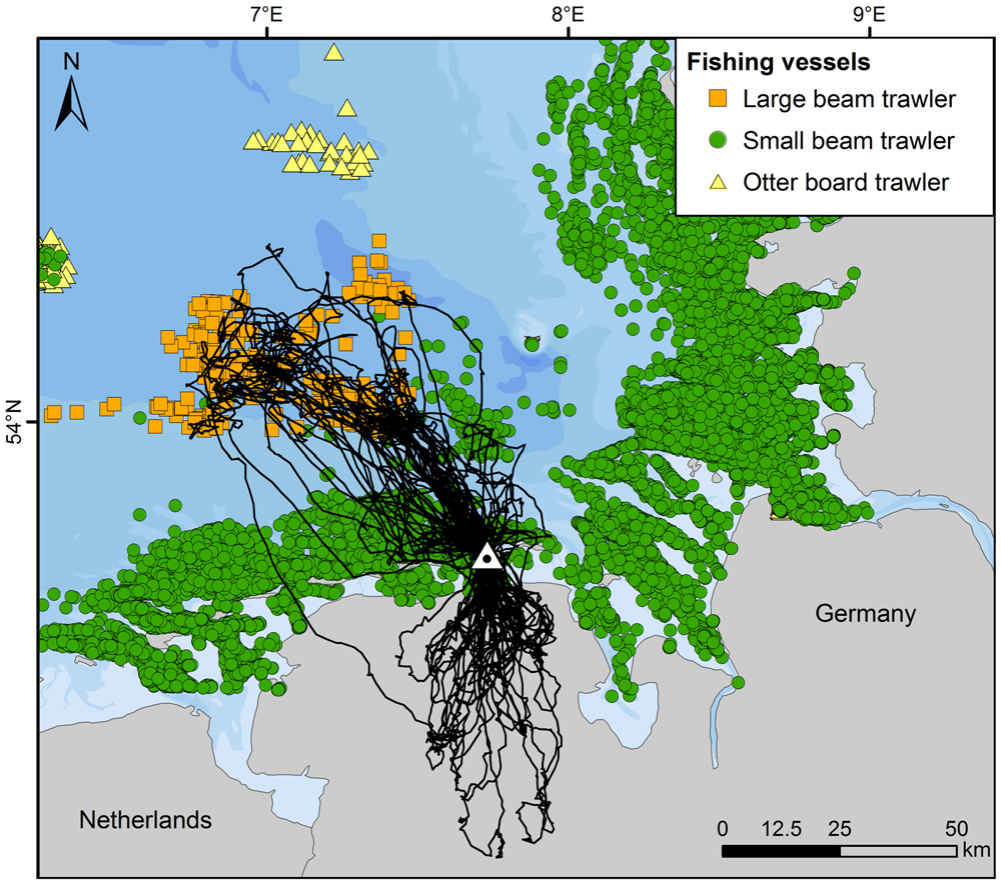
Distribution of fishing vessels and lesser black-backed gull tracks from 17 May to 4 June 2010. Black lines show all flight tracks of lesser black-backed gulls as shown in Fig 1. The location of the colony on the island of Spiekeroog is indicated by a triangle. Areas with water depth <5 m represent the maximum extension of tidal flats during low tide.

The flight patterns of lesser black-backed gulls at sea overlapped substantially with fishing-vessel distribution. While otter board trawlers were operating outside the foraging range of the gulls, both small and large beam trawlers fished within the foraging range (Fig 6). Small beam trawlers, fishing mainly for shrimps *Crangon crangon* and mostly at about 2–10 km north of Spiekeroog island, were intensively attended by lesser black-backed gulls, as shown by the flight tracks (Fig 1) and by telescope from the dunes near the colony. Further offshore, at distances of 30–65 km off the islands, the gulls’ flight tracks responded strongly to the presence of large beam trawlers fishing for flatfish, as shown in Fig 6 and illustrated by the ARS patterns (as e.g. shown for one bird in Fig 3). Dedicated ship surveys run by another project in the same area confirmed that lesser black-backed gulls attended large beam trawlers in high numbers (FTZ unpubl. data).

## Discussion

### Terrestrial and marine foraging areas

Lesser black-backed gulls targeted both marine waters north of the island and inland areas. This is in accordance with recent studies in other parts of the Wadden Sea coast [12, 13]. However, they appeared to ignore the tidal flats of the Wadden Sea, possibly because of competition with a variety of other bird species [8, 43]. The extents to which sea and land areas were visited varied among the eight individuals. In our study, lesser black-backed gulls foraging over the open sea predominantly consumed discarded fish and swimming crabs. However, it should be borne in mind that pellets and faeces may under-represent easily-digestible components and over-represent less-digestible matter, and that prey items have different biomasses, energy densities and assimilation efficiencies (reviewed in [44]). In contrast, while foraging inland, the gulls consumed mostly insects, earthworms and small mammals. Although the sample size of pellets in this study was low, the results were confirmed by findings from other pellet collections at the same colony in 2009 and 2012, and at the neighbouring island of Norderney [9]. The flexibility in feeding ecology of lesser black-backed gulls has been demonstrated by [45] who showed that lesser black-backed gulls can exist and breed successfully on a totally terrestrial diet based on earthworms from pastures.

The radii of the ARS zones and their distances to the colony were significantly larger at sea, at all three scales of ARS. This matches with the general search and foraging behaviours of lesser black-backed gulls. On land, gulls search for a spot (land parcel) where they usually stay for a while and in which they walk or fly very short distances to detect and catch their prey, before moving to another spot to start feeding again. At sea, gulls usually search for prey at longer distance per time rates, because the prey is not aggregated in small units [46] like land parcels, which are either suitable or unsuitable, but with similar food availabilities throughout each specific land parcel. Sea trips were also more complex with respect to the areas covered and trip durations.

Time of day presents an interesting difference between foraging trips at sea and on land; food from terrestrial areas was only taken during daylight periods, while discards from trawlers were targeted at any time of day or night. The observed periods with intense and less intense flight activity at night fit well a wait-and-feed strategy when attending fishing vessels that regularly discard fish and invertebrates. Lighting from fishing boats is apparently sufficient to enable them to be located by lesser black-backed gulls and to allow them to feed on discarded items near the boat. This artificially-illuminated surplus food thus provides the gulls with an extra source of nutrients that allows them to feed opportunistically on a wider variety of prey.

### Importance of anthropogenic food sources

Lesser black-backed gulls from Spiekeroog made intensive use of the anthropogenic landscape and seascape. At sea, fish from trawlers were an important part of the diet. The two main vessel types operating within the foraging range of lesser black-backed gulls from Spiekeroog Island, small beam trawlers fishing for shrimps and large beam trawlers fishing for flatfish, are known to produce the highest discard rates of all German fisheries in the North Sea [47, 48]. The main fish species discarded by flatfish fisheries are plaice (*Pleuronectes platessa*) and dab (*Limanda limanda*), followed by whiting (*Merlangius merlangus*) and grey gurnard [49]. Beam trawlers targeting shrimps mostly discarded (undersized) shrimps, but also swimming crabs and various fish species. All these fish and invertebrate species are known to be preyed upon by lesser black-backed gulls, indicating the relevance of discards as surplus food. Discarding takes place at regular intervals and several times per day so that birds are provided with food at frequent occasions during the 24 h cycle [50]. On land, gulls principally used two types of farmland habitats: fields with open soil (most abundant and most frequently used), potatoes and summer wheat, and meadows that had been freshly mown or grazed by cattle. The use of pastures by gulls has been reported before [21] and is most likely associated with their high organic-matter content leading to increased availability of invertebrate prey [51]. However, this habitat type was proportionally underused in the current study, probably because of the high availability of pastures. Gulls can find prey much more easily where they have direct access to the soil, for example in patches with low vegetation. This suggests that they are likely to benefit from agricultural intensification associated with regular mowing of pastures, higher overall tilled areas, a reduction in grasslands and higher proportion of corn fields with prolonged access to open soils (e.g. [10, 52]).

As hypothesized, SIA revealed significantly higher δ^15^N and δ^13^C values with increasing time spent at sea. This indicates that the gulls’ short-term diet, reflected in the stable isotopes in blood, was in line with their patterns of habitat use. More time spent at sea was thus related to the ingestion of more marine prey from higher trophic levels (discarded fish) compared with prey from terrestrial sites (invertebrates).

## Conclusions

Lesser black-backed gulls from Spiekeroog employed a dual foraging strategy using both marine and terrestrial habitats, similar to the situation described for black-headed gulls [6]. These data demonstrate that lesser black-backed gulls are not an exclusively or even predominantly marine species during the incubation period, in contrast to the conclusions of previous reports [7, 8]. Possible changes in diet and flight patterns over recent years cannot easily be investigated and are outwith the scope of this paper.

Both marine and terrestrial targets appear to be attractive to lesser black-backed gulls. It is difficult to determine which targets are most beneficial, given that food availability cannot be quantified over such different habitats and large areas. However, both marine and terrestrial prey items are diverse and include components with high energy densities. Tracking of individuals during the incubation period suggested that a combination of both sea and inland trips was a useful strategy.

Although the patterns revealed in this study were remarkably similar among individuals and may thus reflect the behaviour of the colony as a whole, differences between colonies are to be expected. For example, foraging patterns would be expected to differ at Amrum Island in the northeastern Wadden Sea as indicated by [13], where swimming crabs constitute roughly 50% of the diet of lesser black-backed gulls [3] and where both natural and discarded fish are relatively scarce in the diet [8, 9]. The current study may serve as a baseline for investigating the effects of future scenarios with reduced availability of discards associated with changes in EU fisheries policy, possibly leading to shifts in food choice towards pelagic fish and/or swimming crabs at sea, or towards terrestrial food. However, further changes in agricultural practice will lead to increased planting of corn for biogas production and a reduction in intensively-used grassland. It will be interesting to see how lesser black-backed gulls adapt to future man-made changes in both 'natural' and 'artificial' landscapes.

## Supporting Information

S1 Fig Variance in First-Passage Time (variance in log(FPT)), as a function of radius r (km), for all eight lesser black-backed gulls. (A) Large-scale variance, from 1 to 50 km, each 1 km, and (B) nested small-scale variance, from 0.1–10 km, each 0.1 km. Different colours represent the average variance in FPT from the several trips performed by different individuals, with peaks of variance indicating area-restricted search (ARS) behaviour.
(TIF)

S2 Fig Individual flight tracks of all eight lesser black-backed gulls breeding on Spiekeroog. The study period was from 17 May to 4 June 2010. The location of the colony on the island of Spiekeroog in the south-eastern Wadden Sea is indicated by a triangle. Areas with water depth <5 m represent the maximum extension of tidal flats during low tide.
(TIF)
