# Supplementary figures and images for: Terrestrial and Marine Foraging Strategies of an Opportunistic Seabird Species Breeding in the Wadden Sea
Source: PLoS One. 2016 Aug 15;11(8):e0159630. doi: 10.1371/journal.pone.0159630 (PMC4985156; doi:10.1371/journal.pone.0159630)

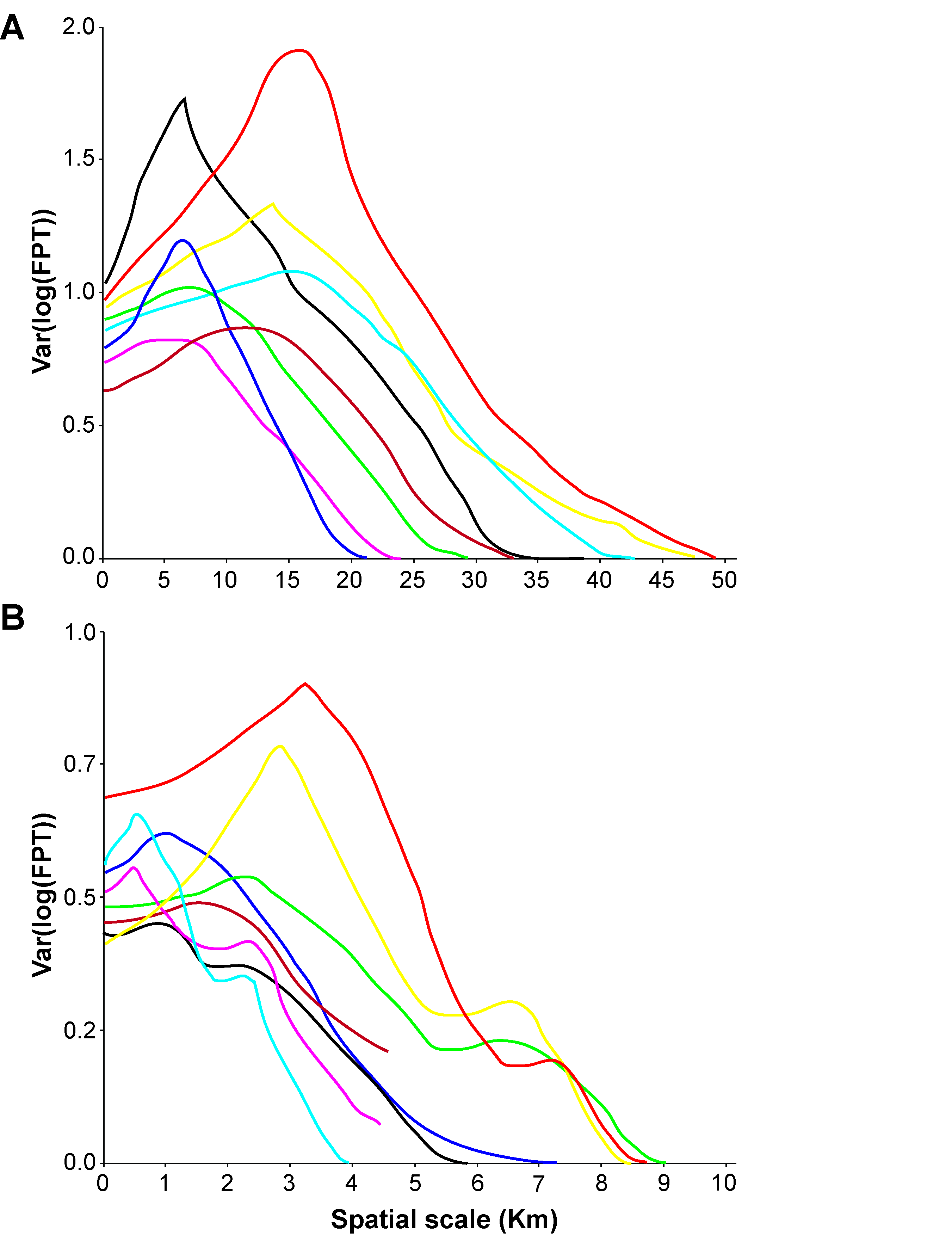

Supplement: S1 Fig — (A) Large-scale variance, from 1 to 50 km, each 1 km, and (B) nested small-scale variance, from 0.1–10 km, each 0.1 km. Different colours represent the average variance in FPT from the several trips performed by different individuals, with peaks of variance indicating area-restricted search (ARS) behaviour. (TIF) [file pone.0159630.s001.tif]

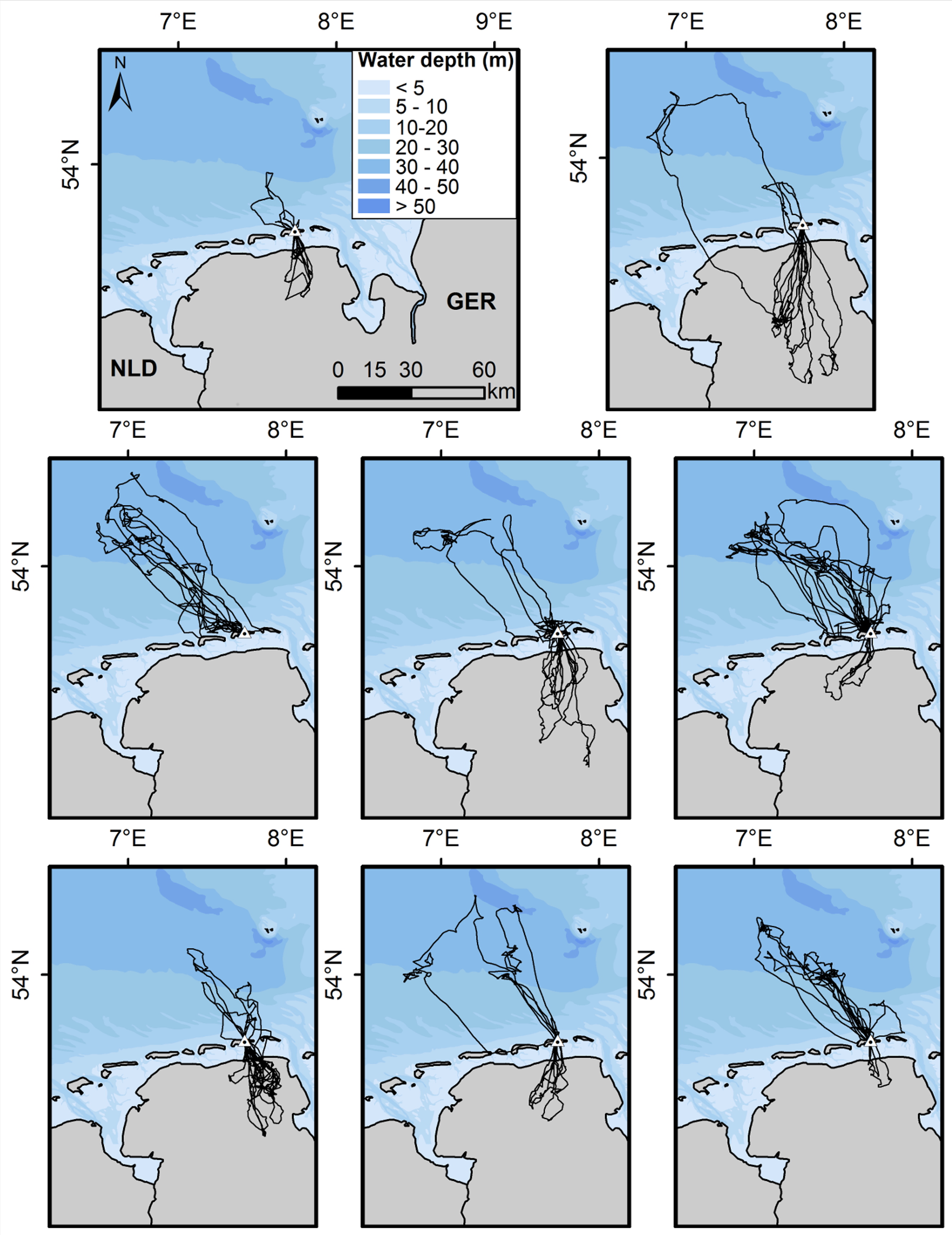

Supplement: S2 Fig — The study period was from 17 May to 4 June 2010. The location of the colony on the island of Spiekeroog in the south-eastern Wadden Sea is indicated by a triangle. Areas with water depth <5 m represent the maximum extension of tidal flats during low tide. (TIF) [file pone.0159630.s002.tif]
